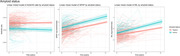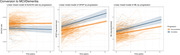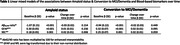# Longitudinal Trajectories of Blood‐based biomarkers for Alzheimer’s Disease in Subjective Cognitive Decline; the SCIENCe project

**DOI:** 10.1002/alz.091234

**Published:** 2025-01-09

**Authors:** Calvin Trieu, Argonde C. van Harten, Mardou S. S. A. van Leeuwenstijn, Lisa‐Marie Schlüter, Inge M.W. Verberk, Lynn Boonkamp, Azzam Aladdin, Charlotte Teunissen, Wiesje M. van der Flier

**Affiliations:** ^1^ Alzheimer Center Amsterdam, Amsterdam UMC, Amsterdam Netherlands; ^2^ Amsterdam Neuroscience, Neurodegeneration, Amsterdam Netherlands; ^3^ Neurochemistry Laboratory, Amsterdam Neuroscience, Program Neurodegeneration, Amsterdam UMC, Amsterdam Netherlands

## Abstract

**Background:**

Blood‐based biomarkers are increasingly able to identify Alzheimer’s Disease pathology in the preclinical stages of the disease. These biomarkers hold promise to study development and progression of the disease. Our aim is to investigate the longitudinal trajectories of Aβ_42/40_ ratio, glial fibrillary acidic protein (GFAP), and neurofilament light (NfL) in individuals with subjective cognitive decline (SCD).

**Method:**

From the SCIENCe cohort, we selected 298 individuals with SCD (age: 61.7±8.1), of whom 80 were amyloid positive (A+; 65.2±7.2) and 218 were amyloid negative (A‐; 60.3±8.0). Amyloid status was assessed using amyloid PET scans or CSF AD biomarkers. Participants underwent annual assessments to evaluate clinical progression to MCI or AD dementia (conversion: n=33). Plasma biomarkers, including Aβ_42/40_ ratio, GFAP, and NfL (pTau217‐pending analysis), were assessed approximately every two years with a mean follow up of 5.3±2.7 years on the SiMoA platform (Quanterix; total sample number: n=819). We used linear mixed models to examine associations between amyloid status and biomarker changes over time, with terms for amyloid status, time, and their interactions. Next, we examined associations between progression to MCI/dementia and biomarker changes over time, with terms for conversion, time, and their interactions.

**Result:**

Aβ_42/40_ was lower in SCD A+ compared to SCD A‐ at baseline (p<0.001), but did not change over time in either group. GFAP and NfL were higher at baseline in SCD A+ (p<0.001; p=0.001). Both increased over time, but only NfL showed a more pronounced increase in SCD A+ compared to SCD A‐ (p=0.006). Aβ_42/40_ was lower in converters than non‐converters at baseline (p<0.001), with no longitudinal differences. NfL and GFAP were higher at baseline in converters (p<0.001; p=0.001), and both displayed a trend toward greater increase over time in converters compared to non‐converters (p=0.059; p=0.095).

**Conclusion:**

Only NfL had a more pronounced increase over time in SCD A+ compared to SCD A‐, whereas all biomarkers differed between the groups at baseline. This suggests that Aβ_42/40_ may have already reached a plateau in SCD A+, while the neurodegeneration marker NfL continues to increase beyond what can be attributed solely to the aging process after the initial SCD diagnosis.